# Safety and efficacy of endoscopic ultrasound‐guided radiofrequency ablation for pancreatic neuroendocrine neoplasms: Systematic review and meta‐analysis

**DOI:** 10.1111/den.14681

**Published:** 2023-10-11

**Authors:** Tawfik Khoury, Wisam Sbeit, Pietro Fusaroli, Davide Campana, Nicole Brighi, Bertrand Napoleon, Andrea Lisotti

**Affiliations:** ^1^ Department of Gastroenterology Galilee Medical Center Nahariya Israel; ^2^ Azrieli Faculty of Medicine Bar‐Ilan University Safed Israel; ^3^ Gastroenterology Unit, Hospital of Imola University of Bologna Bologna Italy; ^4^ Department of Experimental, Diagnostic and Specialty Medicine Sant'Orsola‐Malpighi University Hospital, ENETS Center of Excellence Bologna Italy; ^5^ Department of Medical Oncology IRCCS Romagna Institute for the Study of Tumors “Dino Amadori” Meldola Italy; ^6^ Department of Gastroenterology Jean Mermoz Private Hospital, Ramsay Health Lyon France

**Keywords:** efficacy, meta‐analysis, neuroendocrine tumor, pancreas, safety

## Abstract

**Objective:**

Endoscopic ultrasound‐guided radiofrequency ablation (EUS‐RFA) has been constantly increasing, particularly in the treatment of pancreatic neuroendocrine neoplasms (pNENs). While emerging data in this field are accumulating, we aimed to assess the pooled efficacy and safety of EUS‐RFA for pNENs.

**Methods:**

The PubMed/Medline, Embase, and Cochrane Library databases search was conducted to identify studies reporting EUS‐RFA of pNENs with outcomes of interest (efficacy and safety). The primary outcome was radiological response. Efficacy was assessed by the pooled clinical response rate, whereas safety was assessed by the pooled adverse events (AEs) rate. Heterogeneity was assessed using *I* ^2^. Pooled estimates and the 95% confidence interval (CI) were calculated using a random‐effect model.

**Results:**

Eleven studies involving 292 patients were included. The pooled technical success rate was 99.2% (95% CI 97.9–99.9%), with no heterogeneity. The pooled complete radiological response was 87.1% (95% CI 80.1–92.8%). The pooled partial response was 11.4% (95% CI 6.2–18.1%). The pooled clinical response rate for functional pNENs was 94.9% (95% CI 90.7–97.9%), with no heterogeneity. The pooled incidence of AEs was 20.0% (95% CI 14.0–26.7%); most AEs were mild to moderate in grade, while the pooled incidence of severe AEs was 0.9% (95% CI 0.2–2.3%). The most common AEs were transient mild abdominal pain in 19 patients (6.5%), and mild to moderate pancreatitis in 23 patients (7.9%). No cases of mortality were reported.

**Conclusion:**

Endoscopic ultrasound‐guided radiofrequency ablation resulted on a feasible approach for pNENs treatment, with excellent technical success, high radiological and clinical response, and acceptable AE rate.

## INTRODUCTION

Diagnoses of pancreatic neuroendocrine neoplasms (pNENs) have been increasing over the last years.^1^ Functioning pNENs (F‐pNENs) are frequently detected at early stages due to the occurrence of a clinical syndrome consequent to hormone secretion, leading to prompt investigation. Conversely, nonfunctioning pNENs are asymptomatic and tend to be diagnosed at later stages when they have already grown into larger lesions.^2^ Surgical intervention has traditionally been the primary treatment for these tumors. However, in the past decade advancements in abdominal imaging techniques, such as endoscopic ultrasound (EUS), have led to earlier detection of nonfunctioning pNENs, particularly smaller ones.^2^ This issue represents a therapeutic challenge, as pancreatic surgery, the main therapeutic option for pNENs, is associated with significant morbidity rates and adverse events (AEs).^3^ Radiofrequency ablation (RFA) has been increasingly used for the treatment of solid tumors.^4^, ^5^ Recently, the application of thermal injury or substance injection has been implemented through EUS‐guided treatment.^6^, ^7^ EUS‐RFA is one of the most promising treatment options for pancreatic tumors, especially for pNENs, as RFA induces a tumor mass thermal necrosis that carries low peri‐procedural AEs. Moreover, a previous study has shown that RFA ablation induces the development of a T‐cell‐mediated immune reaction against tumor antigens.^8^ The implementation of EUS‐RFA for pNENs treatment was reported only a few years ago,^9^ and recently, an increasing number of articles in this field have been published. Herein, we provide an updated systematic review and meta‐analysis of all studies addressing the use of EUS‐RFA in pNENs. The primary aim of our study was to evaluate the pooled efficacy (defined as complete radiological response) of EUS‐RFA for the treatment of pNENs; the pooled safety and clinical response rate in F‐pNENs were the secondary objectives.

## METHODS

This systematic review and meta‐analysis was performed in agreement with PRISMA guidelines.^10^

### Search strategy

A bibliographic search was conducted in PubMed/Medline, Embase, and the Cochrane Library databases (limited to English language) independently by two authors (A.L. and T.K.) at the end of April 2023, using the following search string: “(“EUS” OR “EUS‐guided” OR “Endosonography”[Mesh] OR “Endoscopic ultrasound”) AND (ablation OR ablative OR radiofrequency) AND (neuroendocrine OR insulinoma OR endocrine).” The literature search was integrated by additional database evaluations (Google Scholar) and by checking the reference list of all relevant studies on this topic. Prospective or retrospective studies, conference proceedings, and abstracts were considered. In case of overlapping publications on the same population, the most recent reference was included.

### Selection criteria

Studies included in this meta‐analysis were original studies that met the following inclusion criteria: (i) patients: adult patients with pNENs; (ii) intervention: EUS‐RFA; (iii) comparator: none; and (iv) outcomes: the primary outcome was radiological response (complete, partial, and nonresponse), whereas secondary outcomes were the rate of AEs, technical and clinical successes, as clinical response was evaluated in patients with F‐pNENs. We excluded: (i) case series <5 patients; (ii) studies not reporting any of the outcomes; and (iii) studies evaluating other locoregional ablative techniques (i.e. percutaneous ultrasound‐guided RFA, injection therapies, etc.).

### Quality assessment

Quality assessment of retrieved references was independently performed by two authors (A.L. and T.K.) according to the Newcastle–Ottawa scale for nonrandomized studies. Any discordance was reevaluated and supervised by a third author (B.N.).

### Data extraction

For each study the following data were collected: first author's name; year of publication; study population size, gender, and age; number of pNENs; type of pNENs (functioning or nonfunctioning); tumor size (mean size in mm) and location within the pancreas; number of RFA sessions; number of EUS‐RFA applications; type of EUS‐RFA probe; EUS‐RFA power; complete radiological response rate; partial radiological response rate; nonresponse rate; clinical response rate in case of F‐pNENs; incidence and severity of AEs; mortality; and follow‐up duration.

### Outcomes definitions

The primary outcome was efficacy as assessed by radiological response: (i) complete radiological response was defined as complete disappearance of enhanced solid pancreatic neoplasm or evidence of complete colliquative necrosis on cross‐sectional imaging during follow‐up; (ii) partial response was defined as the reduction of tumor size between 75% and 95%; and (iii) nonresponse was defined as a lack of volume reduction or volume reduction <75%.

Secondary outcomes were: (i) AEs rate, calculated as the amount of AE/number of patients who underwent EUS‐RFA. AEs were graded according to the American Society for Gastrointestinal Endoscopy lexicon;^11^ (ii) technical success rate, defined as the successful performance of EUS‐RFA (needle insertion and feasibility of ablation); and (iii) clinical success rate, assessed in patients with F‐pNENs and based on the disappearance of a secretory syndrome (i.e. hypoglycemia) after EUS‐RFA.

### Statistical analysis

Study outcomes were pooled through a random‐effects model based on the DerSimonian and Laird test, and results were expressed in terms proportion (%) and 95% confidence interval (CI). Heterogeneity was assessed through *I* ^2^ tests: *I* ^2^ <30% was considered as a low level of heterogeneity, *I* ^2^ between 30% and 60% as moderate heterogeneity, while *I* ^2^ >60% was interpreted as a high level of heterogeneity. Any potential publication bias was verified through visual assessment of funnel plots and using the Egger's test.

Sensitivity analysis was performed on the primary outcome measure on study design (prospective or retrospective), study population, pNEN secretory status, mean pNEN size, pNEN location, and RFA power setting. Statistical analysis was performed with MedCalc Statistical Software version 20.115 (MedCalc Software, Ostend, Belgium; https://www.medcalc.org; 2022). A two‐tailed *P*‐value < 0.05 was considered statistically significant.

## RESULTS

### Quality assessment

Five studies showed an overall low quality, while the remaining six a medium quality. The mean Newcastle–Ottawa Score was 4.9 (range, 4–6). Quality assessment is detailed in Table S1.

### Study characteristics

**Figure 1:**
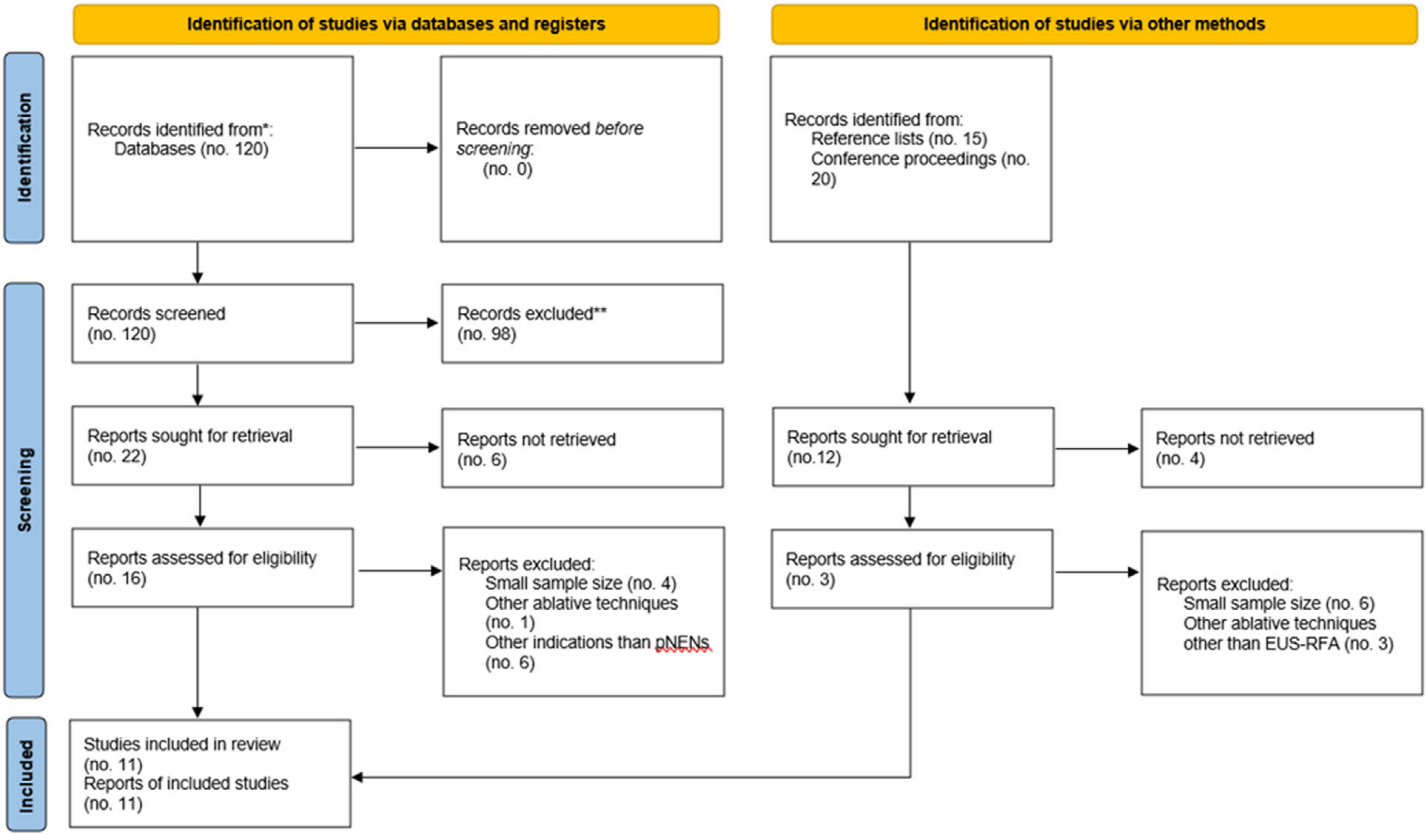
PRISMA flow diagram of the study. *Consider, if feasible to do so, reporting the number of records identified from each database or register searched (rather than the total number across all databases/registers). **If automation tools were used, indicate how many records were excluded by a human and how many were excluded by automation tools. From: Page *et al*.^10^ For more information, visit: http://www.prisma‐statement.org/. EUS‐RFA, endoscopic ultrasound‐guided radiofrequency ablation; pNEN, pancreatic neuroendocrine neoplasm.

The literature search through Medline resulted in 120 full‐text articles; the other 35 references were retrieved through the evaluation of major gastroenterology conference proceedings. Figure 1 summarizes the literature search according to the PRISMA reporting form. After a preliminary screening of titles, 34 publications were selected to be reviewed as full text, and 19 were assessed for eligibility. Finally, 11 studies^9^, ^12^, ^13^, ^14^, ^15^, ^16^, ^17^, ^18^, ^19^, ^20^, ^21^ were included in the qualitative and quantitative analysis. Among them, 10 studies were published as full text, while the remaining one was presented as an abstract at international conferences. Four studies (36.4%) were designed as a prospective design; four studies (36.4%) were multicentric. Studies' characteristics are summarized in Table 1.

**Table 1:** Study characteristics of all studies included in the meta‐analysis

	Choi *et al*.^12^	Barthet *et al*.^9^	Oleinikov *et al*.^13^	de Nucci *et al*.^14^	Marx *et al*.^15^	Younis *et al*.^17^	Marx *et al*.^16^	Crinò *et al*.^19^	Napoléon *et al*.^18^	Rizzatti *et al*.^21^	Borrelli de Andreis *et al*.^20^
Year	2018	2019	2019	2020	2022	2022	2022	2023	2023	2023	2023
Type	Prospective	Prospective	Retrospective	Prospective	Retrospective	Prospective	Retrospective	Retrospective	Retrospective	Prospective	Retrospective
Patient number	8	12	18	10	27	7	7	89	64	56	10
Male (*N*)	4	7	10	5	14	4	1	27	NR	NR	3
Lesion number	8	14	27	11	27	7	7	–	64	56	10
Mean age (years)	55.0	59.9	60.4	78.6	65.0	67.4	66.0	55.1	NR	–	67.1
Type of pNENs											
Insulinoma	1	0	7	0	0	1	7	89	16	24	10
NF‐pNENs	7	14	11	11	27	6	0	0	48	32	0
Grade (*N*)											
G1	1	12	15	10	25	5	4	66	48	NR	9
G2	1	0	NR	0	NR	1	1	3	4	NR	1
G3	0	0	3	0	NR	0	NR	0	3	NR	0
Unknown	6	0	1	0	2	0	2	20	9	–	–
Locations	–	–	–	–	–	–	–	–	NR	NR	–
Head	3	3	15	3	8	2	1	34	–	–	3
Neck	0	0	0	0	0	0	3	0	–	–	0
Body	5	6	8	5	3	4	2	39	–	–	3
Tail	0	5	2	3	11	1	0	16	–	–	4
Lesion mean size (mm)	19.25 (8–28)	13.1 (10–20)	14.8 (12–19)	14.5 (9–20)	14.0 (7–25)	10.7 (6–18)	13.3 (8–20)	13.4	15.0 (5–30)	NR	11.9
RFA power (W)	50	50	50	20	50	50	50	10–50	NR	NR	20–50
RFA sessions (*N*)	14	12	18	10	31	7	7	NR	NR	NR	11
RFA applications (*N*)	65	NR	3–10 each session	23	1–5 each session	22	1–5 each session	283	NR	1–3	1–2
Size of RFA needle (G)	18–19	19	19	19	19	19	19	18–19	19	19	19
Proximity to PD (mm)	NR	2	NR	2	NR	NR	NR	>2 (53) ≤2 (21)	NR	NR	NR
Antibioprophylaxis (*N*)	Yes	Yes	Yes	Yes	Yes	NR	Yes	61	NR	Yes	Yes
Rectal NSAIDs (*N*)	NR	No	NR	Yes	No	Yes	Yes	74	NR	Yes	No
Prophylactic PD stent	NR	NR	NR	NR	No	NR	No	NR	NR	No	No
Radiological follow‐up (months)	13 (8–30)	12	8.7 (2–21)	12	15.7 (2–41)	7	20.3 (3–38)	23 (14–31)	12	12	12
Clinical follow‐up (months)	13 (8–30)	NR	9.7 (3–21)	NR	NR	12	21 (3–38)	NR	12	12	12
Mortality	None	None	None	None	None	None	None	None	None	None	None

NF‐pNEN, nonfunctioning pancreatic neuroendocrine neoplasm; NR, not reported; NSAID, nonsteroidal anti‐inflammatory drug; PD, pancreatic duct; pNEN, pancreatic neuroendocrine neoplasm; RFA, radiofrequency ablation.

### Primary outcome (pooled efficacy)

**Figure 2:**
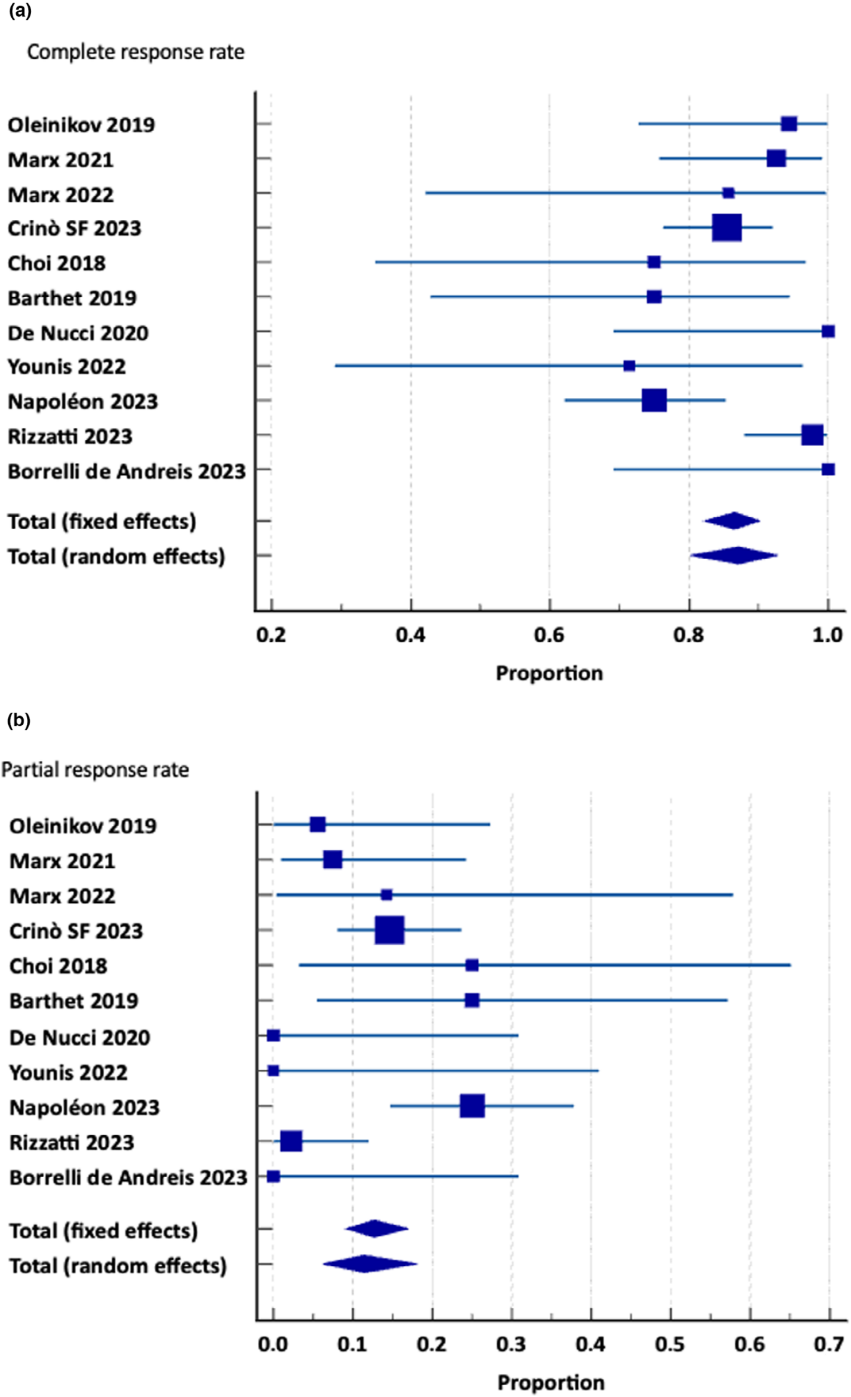
(a) Complete radiological response. (b) Partial radiological response.

The pooled complete radiological response (11 studies; 292 patients) was 87.1% (95% CI 80.1–92.8%), with moderate heterogeneity *I* ^2^ 55.7%, while pooled partial response was 11.4% (95% CI 6.2–18.1%), with moderate heterogeneity *I* ^2^ 54.8%. Figure 2 shows forest plots for pooled radiological response.

### Secondary outcomes

**Figure 3:**
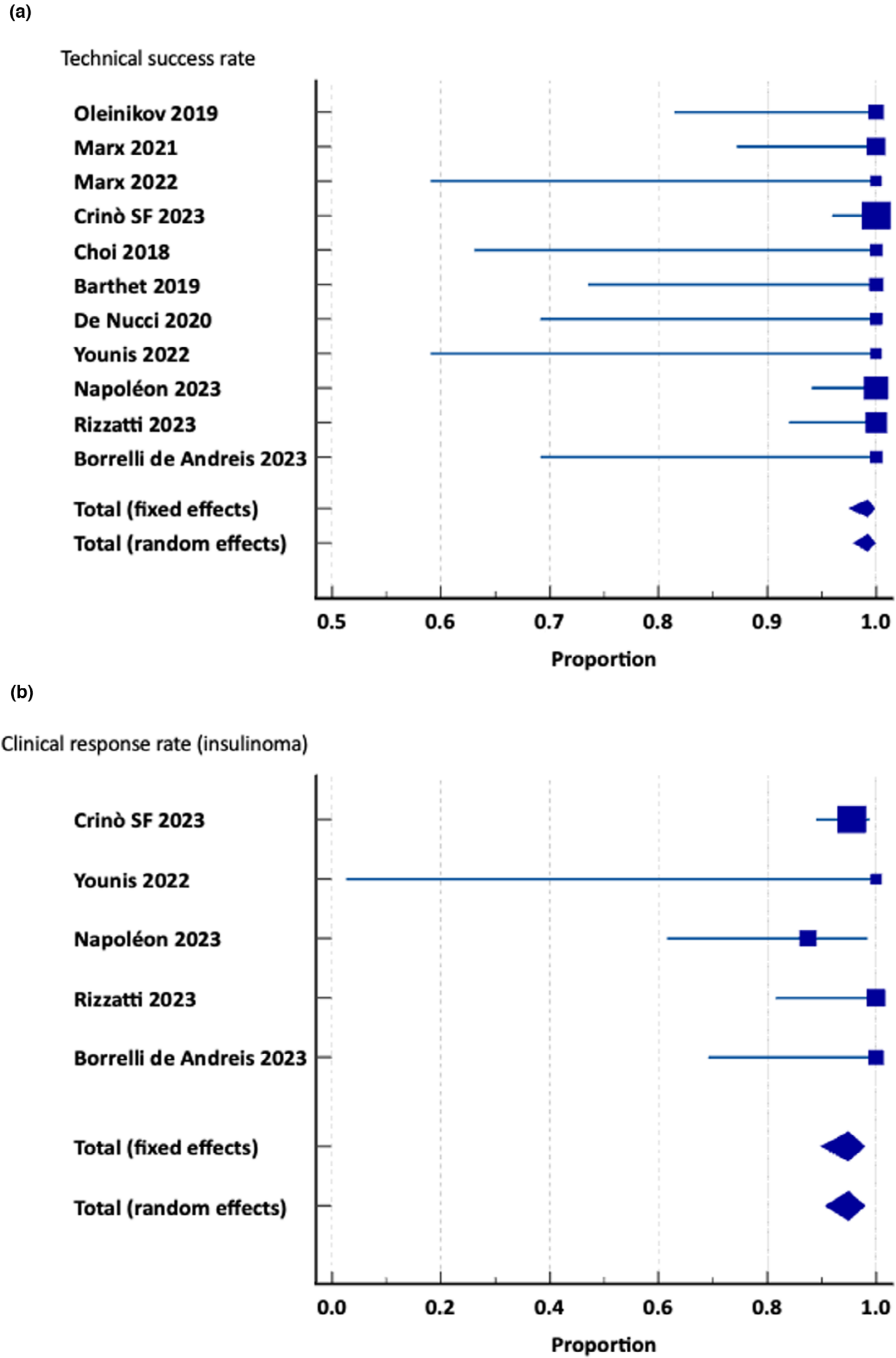
(a) Technical success rate. (b) Clinical response rate.

The pooled clinical response rate (five studies; 134 patients) was 94.9% (95% CI 90.7–97.9%), with no heterogeneity (*I* ^2^ 0.0%). The technical success rate was reported in 11 studies (292 patients); the pooled technical success rate was 99.2% (95% CI 97.9–99.9%), with no heterogeneity (*I* ^2^ 0.0%). Figure 3 shows forest plots for pooled technical and clinical success rates.

### Safety

**Figure 4:**
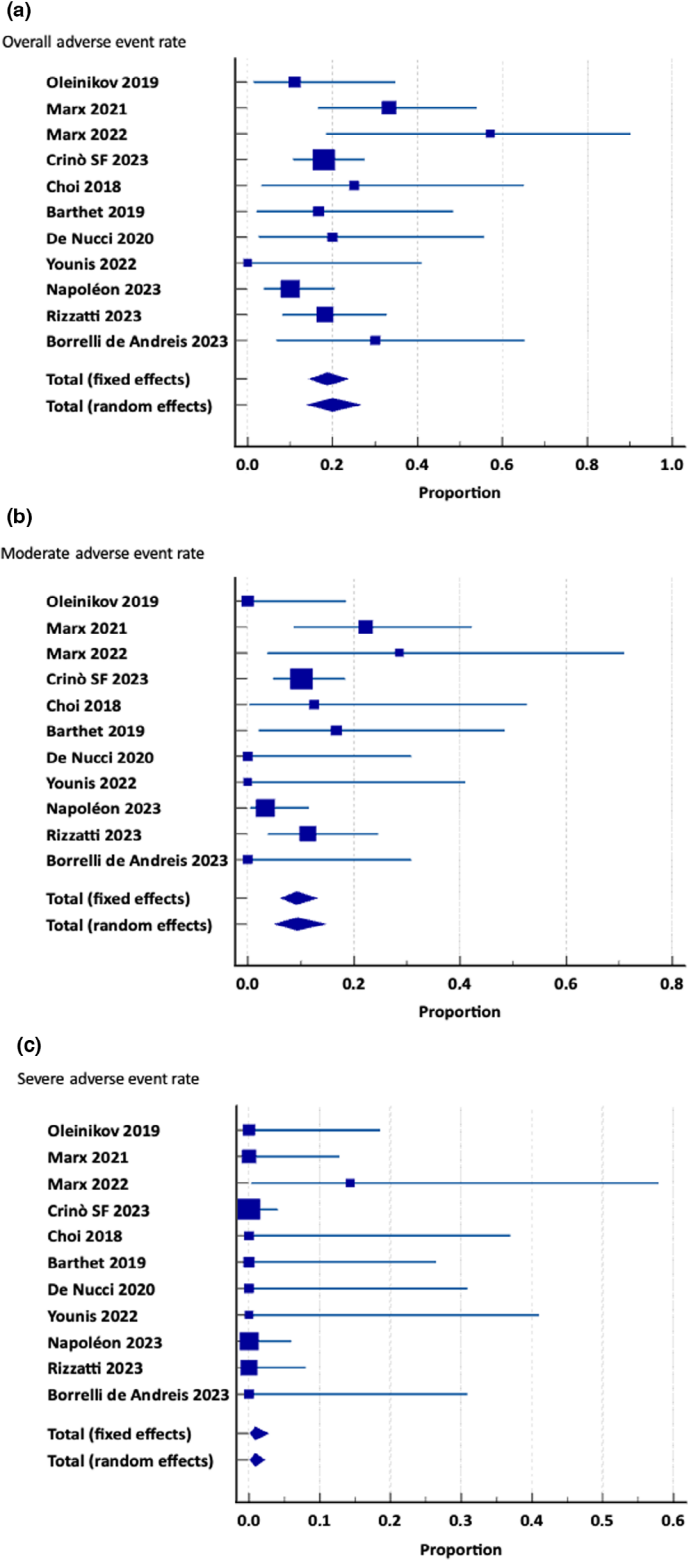
(a) Overall adverse events rate, (b) moderate adverse events rate, (c) severe adverse events rate.

The pooled rate of AEs (11 studies; 292 patients) was 20.0% (95% CI 14.0–26.7%), with moderate heterogeneity (*I* ^2^ 37.6%); the pooled rate of severe AEs (11 studies; 292 patients) was 0.9% (95% CI 0.2–2.3%), with no heterogeneity (*I* ^2^ 0.0%). Most of the studies had mild–moderate procedure‐related AEs: transient mild abdominal pain occurred in 19 patients,^12^, ^14^, ^15^, ^16^, ^18^, ^19^ mild–moderate pancreatitis in 23 patients,^12^, ^13^, ^15^, ^16^, ^17^, ^18^, ^19^ mild peri‐procedural bleeding in two patients,^15^ severe pancreatitis in seven patients,^9^, ^15^, ^16^, ^18^ pancreatic duct stenosis in one patient,^9^ diabetes mellitus in one patient,^19^ and spleen hematoma in one patient treated with conservative management and surgical intervention.^19^ Notably, in the study by Crinò *et al*.,^19^ in eight out of the nine patients who had post‐EUS‐RFA pancreatitis, the distance between the lesion and the main pancreatic duct was ≤2 mm. Figure 4 shows forest plots for the incidence of overall AEs, and moderate and severe AEs (Table 2).

**Table 2:** Pooled estimates

	Estimates random‐effect model	Heterogeneity	Publication bias Egger's test
Technical success rate (11 studies; 292 patients)	99.2% (97.9–99.9%)	*I* ^2^ 0.0%	<0.001
Complete response rate (11 studies; 292 patients)	87.1% (80.1–92.8%)	*I* ^2^ 55.7%	0.230
Partial response rate (11 studies; 292 patients)	11.4% (6.2–18.1%)	*I* ^2^ 54.8%	0.460
Clinical response rate (5 studies; 134 patients)^†^	94.9% (90.7–97.9%)	*I* ^2^ 0.0%	0.800
Adverse event rate (11 studies; 292 patients)	20.0% (14.0–26.7%)	*I* ^2^ 37.6%	0.290
Moderate AE rate (11 studies; 292 patients)	9.4% (5.1–14.7%)	*I* ^2^ 41.3%	0.120
Severe AE rate (11 studies; 292 patients)	0.9% (0.2–2.3%)	*I* ^2^ 0.0%	0.002

^†^ Clinical response rate was assessed in patients with functioning pancreatic neuroendocrine neoplasms.

AE, adverse event.

### Sensitivity analysis

As reported in Table 3, the sensitivity analyses was restricted to study design, study population, pNEN secretory status, mean pNEN size, and power RFA settings, which confirmed the main outcome results with moderate heterogeneity in all but two conditions. Low heterogeneity was observed for F‐pNENs (pooled complete radiological response 87.5% [95% CI 77.5–94.8%]; *I* ^2^ 19.9%), small (<15 mm) pNEN size (pooled complete radiological response 87.9% [95% CI 81.5–93.0%]; *I* ^2^ 20.6%), and in case of pNENs located in the head or uncinate process (pooled complete radiological response 89.6% [95% CI 75.5–98.0%]; *I* ^2^ 0.0%).

**Table 3:** Sensitivity analysis for main outcome measure

	Pooled estimates (95% CI) random‐effect model	Heterogeneity
Study design		
Retrospective studies (7 studies; 218 patients)	85.3% (77.9–91.4%)	*I* ^2^ 40.2%
Prospective studies (4 studies; 74 patients)	89.0% (72.4–98.5%)	*I* ^2^ 65.9%
Study population		
<25 patients (6 studies; 72 patients)	86.6% (75.8–94.5%)	*I* ^2^ 35.3%
≥25 patients (5 studies; 220 patients)	87.7% (76.9–95.4%)	*I* ^2^ 77.4%
Secretory status		
Nonfunctioning NENs (3 studies; 49 patients)	89.2% (74.3–98.2%)	*I* ^2^ 48.2%
Functioning NENs (3 studies; 109 patients)	87.5% (77.5–94.8%)	*I* ^2^ 19.9%
Mean NEN size		
<15 mm (8 studies; 180 patients)	87.9% (81.5–93.0%)	*I* ^2^ 20.6%
≥15 mm (3 studies; 112 patients)	84.3% (61.1–98.0%)	*I* ^2^ 85.2%
NEN location		
Head/uncinate (2 studies; 25 patients)	89.6% (75.5–98.0%)	*I* ^2^ 0.0%
Body/tail (7 studies; 216 patients)	85.3% (77.0–92.1%)	*I* ^2^ 51.0%
RFA power setting		
<50 W (3 studies; 109 patients)	92.4% (79.8–99.1%)	*I* ^2^ 52.7%
50 W (8 studies; 183 patients)	84.6% (74.6–92.4%)	*I* ^2^ 60.9%

CI, confidence interval; NEN, neuroendocrine neoplasm; RFA, radiofrequency ablation.

### Publication bias

A potential publication bias was observed for the technical success rate (Egger's test: intercept −1.03; 95% CI −1.03 to −1.02; *P* < 0.001) and incidence of severe AEs (Egger's test: intercept 1.43; 95% CI 0.68–2.17; *P* = 0.002). No publication bias was observed for complete and partial response rates, clinical response rate, or incidence of AEs (Table 2).

## DISCUSSION

Diagnoses of pNENs have increased in the last decade due to the widespread availability of cross‐sectional abdominal imaging.^22^ pNENs account for approximately 8–10% of all pancreatic neoplasms.^23^ Traditionally, pNENs were treated with pancreatic surgery, which is associated with relevant comorbidities.^24^, ^25^, ^26^ The features of related comorbidities were previously recapitulated in a systematic review and meta‐analysis by Jilesen *et al*.,^3^ reporting the occurrence of pancreatic fistulas in 14–58% cases, postoperative hemorrhage in 1–6%, and delayed gastric emptying in 5–18%, with an overall pooled in‐hospital mortality of 4–6%. Therefore, in the last few years locoregional treatment options including EUS‐guided ethanol injection and EUS‐guided RFA^27^ have been implemented for the treatment of pNENs.

Herein, we performed an updated systematic review and meta‐analysis of all studies reporting EUS‐RFA for pNENs in the literature (in English). We observed an excellent technical success of EUS‐RFA for pNENs in 99.2% (95% CI 97.9–99.9%) among the 11 studies assessed (292 patients). Similarly, the complete radiological response was high in 87.1% (95% CI 80.1–92.8%). Our data were compatible with a recent review article that showed a technical success of 100%, and complete radiological success of 90%.^28^ Moreover, the clinical success rate in our study, especially in patients with F‐pNENs, was high, 94.9% (95% CI 90.7–97.9%). To date, only few systematic reviews and meta‐analyses assessing EUS‐RFA for pNENs with a relatively low number of patients were published. Imperatore *et al*.^29^ reported results on 61 patients who underwent EUS‐RFA, with an overall effectiveness of 96% on a mean follow‐up period of 11 months, with mild AEs occurring in 13.7%. Armellini *et al*.^30^ reported clinical efficacy of 95.1% (95% CI 91.2–98.9%). The overall AE rate in our meta‐analysis was 20.0% (95% CI 14.0–26.7%). In particular, the rate of severe AEs was very low (0.9% [95% CI 0.2–2.3%]), similar to data published in a previous meta‐analysis of 17.8% (95% CI 9.1–26.4%),^30^ while the mild AE rate was 13.7%.^29^

Notably, our meta‐analysis differs from the aforementioned two meta‐analyses,^29^, ^30^ as it represents an updated meta‐analysis including recent studies with a large number of patients, and excluding case reports and small case series that were instead included in previous meta‐analyses. Indeed, this approach could strengthen our meta‐analysis, as it removes case reports and small case series that could represent a challenge for internal validity evaluation, as suggested by systematic review guidelines.^31^

To date, there is scarce evidence regarding the parameters that may predict EUS‐RFA failure and AEs. In our study we performed a sensitivity analysis for several parameters, including study design, pNEN functional status, pNEN size, and the RFA setting that showed no significant effect of the parameters assessed, except for pNENs smaller than 15 mm, which can be associated with a robust favorable impact of EUS‐RFA. A previous systematic review showed that larger pNEN size was associated with treatment failure, as it was shown that lesions <18 mm at EUS were associated with an excellent response to treatment in 97.1% of cases.^29^

Thus, EUS‐RFA could even be considered as a first therapeutic treatment option for small pNENs. Moreover, in our study we showed that the power setting of the RFA system slightly affected the primary outcome, as a power setting of <50 W had achieved the primary outcome in 92.4% vs. 50 W in 84.6%. Notably, we could not retrieve any previously published data regarding this issue, warranting further studies to precisely address power settings requirements for pNENs. EUS‐RFA is considered a minimally invasive procedure with a good safety profile. In our study we have shown an acceptable AE rate, mostly of mild to moderate severity, and a very low incidence of severe AE rate, and no mortality cases, suggesting an acceptable safety profile of EUS‐RFA in pNENs, especially when comparing it to traditional surgical approaches.

Additionally, the number of RFA sessions and applications were different among the included studies, and this could be attributed to the lesion size. In fact, the bigger the lesion size, the more RFA applications and sessions were needed, as shown in the study by Choi *et al*.,^12^ as compared to the other studies. However, further studies with uniform lesion size, RFA sessions, and application are warranted to precisely assess the impact of the number of RFA sessions and RFA applications on clinical and radiological outcomes.

Our meta‐analysis has some limitations. First, the setting power of the RFA was not standardized, potentially affecting treatment response. Data regarding other variables such as proximity to pancreatic duct, prophylaxis with antibiotics, and pancreatic duct stenting were often lacking; however, some studies adopted those preventive measures, while others did not, and the report on this issue was not consistent in this meta‐analysis, and this could affect data interpretation and results. We have to acknowledge that an abstract (Rizzatti *et al*.,^21^ not peer‐reviewed yet) has been included in the meta‐analysis; however, its prospective multicenter study design has been presented in the ESGE Days Conference as an oral presentation and this, in our opinion, makes it a reliable study to be included in the meta‐analysis.

On the other hand, our study is currently a meta‐analysis reporting data on the largest number of patients, thus supporting the previous published studies, and further augmenting the evidence of EUS‐RFA for the treatment of pNENs. Finally, since most of the included studies had a retrospective design, a potential selection bias cannot be excluded: in fact, most studies included mainly pNENs located in the body or tail of the pancreas, suggesting that small tumors located in the head or uncinate process may have not been treated with EUS‐RFA due to technical issues, such as scope instability or difficulty in identifying an operative window.

In conclusion, EUS‐RFA can be considered an effective and safe, minimally invasive option for the treatment of pNENs. According to the currently available data, it is reasonable to support the use of RFS for small pNEN treatment, regardless of their functional status. Larger series with longer follow‐up are needed to better identify which pNEN patients would benefit from endoscopic treatments and which would be better treated surgically.

## CONFLICT OF INTEREST

Authors declare no conflict of interest for this article.

## FUNDING INFORMATION

None.

## DISCLOSURE

Author B.N. is giving a teaching session for the Taewong Company.

## Supporting information


**Table S1** Quality assessment of included studies according to the Newcastle–Ottawa Scale (NOS) for nonrandomized studies.
