# Supplementary material for: Safety and efficacy of endoscopic ultrasound‐guided radiofrequency ablation for pancreatic neuroendocrine neoplasms: Systematic review and meta‐analysis
Source: Dig Endosc. 2023 Oct 11;36(4):395–405. doi: 10.1111/den.14681 (PMC12136273; doi:10.1111/den.14681)
Supplement: Supplementary file 1 — Table S1 Quality assessment of included studies according to the Newcastle–Ottawa Scale (NOS) for nonrandomized studies. [file DEN-36-395-s001.docx]

**Supplementary table 1.** Quality assessment of included studies according to the Newcastle-Ottawa Scale (NOS) for non-randomized studies

| **Reference** | **Selection** | **Comparability** | **Outcome** | **Overall** |
| --- | --- | --- | --- | --- |
| Choi et al. 2018 | *** | N/A | *** | Medium |
| Barthet M et al. 2019 | *** | N/A | *** | Medium |
| Oleinikov K et al. 2019 | * | N/A | *** | Low |
| De Nucci G et al. 2020 | *** | N/A | *** | Medium |
| Marx M et al. 2021 | * | N/A | *** | Low |
| Younis F et al. 2022 | * | N/A | *** | Low |
| Marx M et al. 2022 | * | N/A | *** | Low |
| Crinò SF et al. 2023 | ** | N/A | *** | Medium |
| Napoléon B et al. 2023 | ** | N/A | *** | Medium |
| Rizzatti G et al. 2023 (ABS) | *** | N/A | *** | Medium |
| Borrelli de Andreis et al. 2023 | * | N/A | *** | Low |

Abbreviation: N/A – not applicable
